# Depression increases the risk of contracting genital herpes and genital wart in U.S. adults: A cross-sectional analysis

**DOI:** 10.1097/MD.0000000000046800

**Published:** 2025-12-26

**Authors:** Hongren Wu, Qiuhong Ye, Pengcheng Sun, Jianjun Zhang, Chuanhui Yan

**Affiliations:** a Shanxi Key Laboratory of Chinese Medicine Encephalopathy, National International Joint Research Center for Molecular Chinese Medicine, Shanxi University of Chinese Medicine, Jinzhong, Shanxi, China; b College of Basic Medical Sciences, Shanxi University of Chinese Medicine, Jinzhong, Shanxi, China.

**Keywords:** depression, genital herpes, genital warts, sexually transmitted infections

## Abstract

Depression is associated with a higher prevalence of self-reported risky sexual behaviors, potentially increasing susceptibility to sexually transmitted infections (STIs). However, epidemiological evidence linking depression to genital herpes or genital warts remains limited. This cross-sectional analysis utilized data from the National Health and Nutrition Examination Survey. Depressive symptoms were assessed using the validated Patient Health Questionnaire-9. STIs were self-reported via audio computer-assisted self-interview in private rooms at the Mobile Examination Center. Multivariable logistic regression models were employed to evaluate associations between depression and genital herpes/warts-related outcomes, supplemented by subgroup analyses to identify vulnerable populations. Adults with depression demonstrated significantly elevated risks of genital herpes (odds ratio = 1.73, 95% confidence interval:1.22–2.45) and genital warts (odds ratio = 1.79, 95% confidence interval:1.36–2.35) compared with nondepressed individuals. Subgroup analyses revealed differential vulnerability across demographic and clinical strata, with females, individuals with postsecondary education, and hypertensive patients exhibiting heightened susceptibility to both infections. Depression was associated with higher odds of genital herpes and genital warts among US adults. These findings underscore the clinical importance of integrating STIs screening into mental health management protocols, particularly among females and individuals with higher educational attainment. Enhanced preventive strategies targeting high-risk sexual behaviors in depressed populations are warranted.

## 1. Introduction

Depression, a globally prevalent mental health disorder affecting an estimated 5% of adults,^[1]^ is associated with persistent low mood and increased engagement in risky sexual behaviors. Studies indicate that individuals with depression report higher rates of risky sexual behaviors compared with nondepressed individuals,^[2]^ with women – particularly those with severe depression – exhibiting the highest risk.^[3]^ Adolescents with severe depressive symptoms also demonstrate elevated likelihood of subsequent risky sexual behaviors,^[4]^ contributing to a heightened risk of sexually transmitted infections (STIs) in this population.^[5]^

Genital herpes and genital warts, 2 STIs strongly linked to risky sexual behaviors, show rising global incidence.^[6]^ Genital herpes, primarily caused by herpes simplex virus type 2 (HSV-2), is characterized by recurrent symptomatic outbreaks and increased biological susceptibility to human immunodeficiency virus (HIV) acquisition. Current estimates indicate 846 million people aged 15 to 49 worldwide live with genital herpes, resulting in annual socioeconomic costs of $35 billion.^[7]^ Genital warts, caused by human papillomavirus (HPV) types 6 and 11 (responsible for >90% of cases^[8]^), manifest through 4 clinical subtypes: condyloma acuminatum (cauliflower-like projections), flat/macular lesions, papular eruptions, and keratotic formations.^[9,10]^ Global prevalence ranges from 0.15% to 0.18%.^[11]^

Though genital warts and herpes seldom lead to life-threatening outcomes,^[10,12]^ the psychological toll of disease-related stigma and associated distress significantly burdens affected individuals.^[13–18]^ Research indicates that patients with these conditions experience higher depression levels compared with those with other STIs.^[19]^

Most research on the association between depression and STIs has mainly focused on HIV, chlamydia, trichomoniasis, and syphilis, with limited attention given to genital herpes and warts. Prospective studies indicate that depression correlates with heightened STI risks, including genital herpes and warts, among US adolescents.^[20]^ Similarly, retrospective cohort studies conducted in Taiwan have demonstrated that patients with depression face a significantly higher risk of developing STIs, including genital warts, in the future.^[21]^ Nevertheless, there are contrasting finding showed no significant difference in the mean IgG antibody titers for HSV-1 and HSV-2 between depressed patients and healthy controls in a retrospective study.^[22]^

This study aimed to analyze National Health and Nutrition Examination Survey (NHANES) data to investigate the potential association between depression and genital herpes or genital warts and identify factors influencing this relationship. The analysis will enable a more accurate assessment of the prevalence of these conditions among individuals with depression and support targeted prevention and screening strategies.

## 2. Methods

### 2.1. Database and study population

The original data required for this study were obtained from the NHANES, which is administered by the National Center for Health Statistics. NHANES conducts comprehensive surveys of the health and nutritional status of adults and children in the United States on a biennial basis. The data collection procedures were approved by the ethics review board of the National Center for Health Statistics, and written informed consent was obtained from all participants. The data are publicly available.

**Figure 1.**
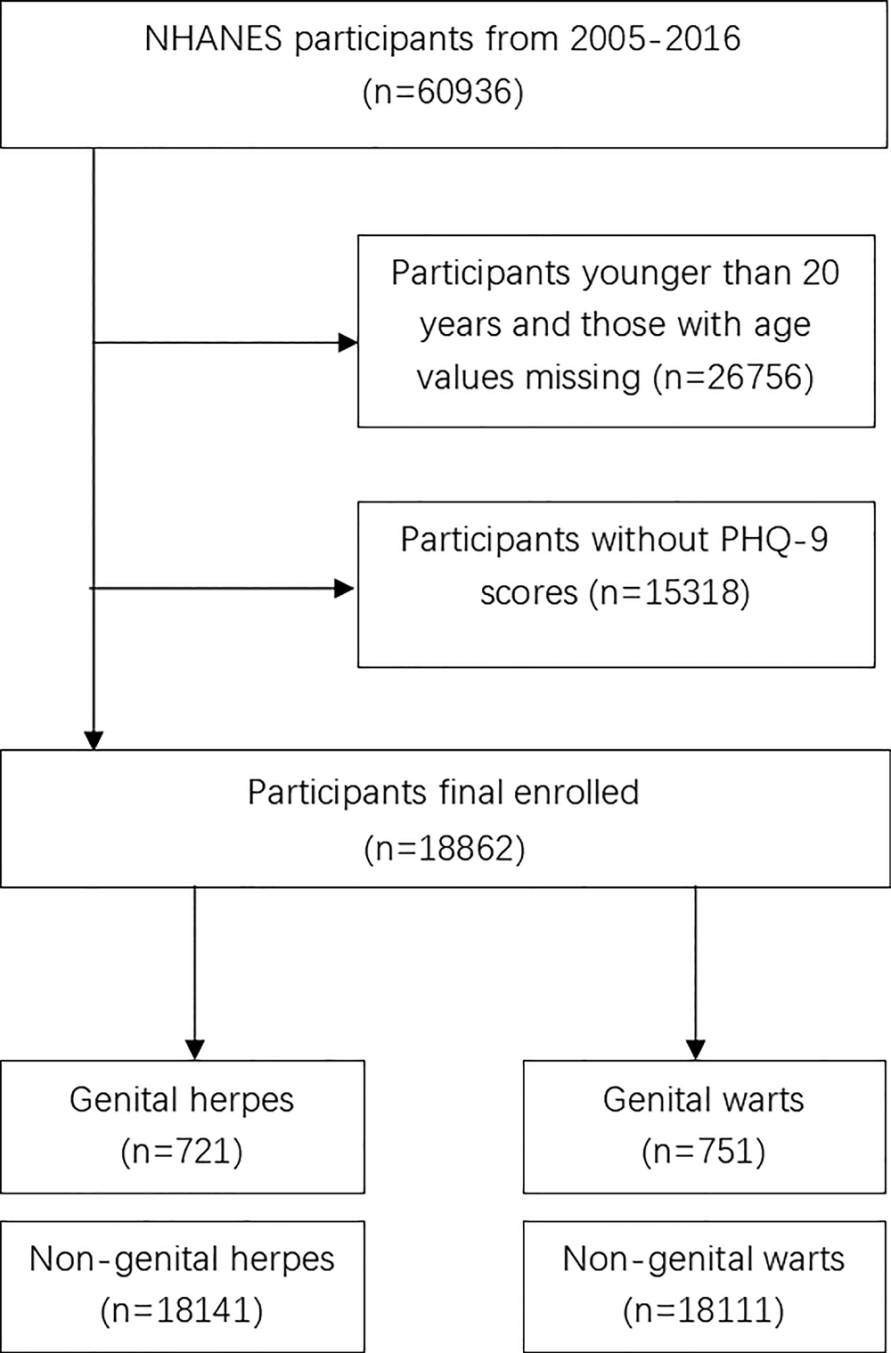
Flow diagram of the selection process for participants. NHANES = National Health and Nutrition Examination Survey.

The data utilized in this study encompassed 6 cycles spanning from 2005 to 2016, incorporating survey data from a total of 60,936 participants. Of these, 26,756 individuals were excluded due to being under the age of 20 or having incomplete age information. An additional 15,318 participants were excluded because of missing Patient Health Questionnaire-9 (PHQ-9) scores, resulting in a final sample size of 18,862 participants. To separate analyses for genital herpes and genital warts, the final sample included 721 cases of genital herpes and 751 cases of genital warts among the 18,862 participants. Figure 1 provides a detailed illustration of the participant selection process. To address missing covariates, multiple imputation was employed to impute missing values, thereby reducing potential bias and preserving statistical power.

### 2.2. Identification of patients with depression

The NHANES employs the PHQ-9 to evaluate depressive symptoms. The PHQ-9 is widely utilized in primary care^[23]^ settings for diagnosing depression by assessing the frequency of depressive symptoms over the past 2 weeks. This screening tool quantifies responses to all 9 items on a 4-point Likert scale, ranging from “not at all” (0) to “nearly every day” (3). Scores are summed, with a range of 0 to 27, where higher scores indicate more severe depressive symptoms. The sensitivity and specificity of this instrument in semi-structured interview research have been reported as 0.88 (95% confidence interval [CI]: 0.83–0.92) and 0.85 (95% CI: 0.82–0.88), respectively. A cutoff score of 10 or higher optimizes combined sensitivity and specificity for detecting major depression.^[24]^ Consequently, in this study, participants with a PHQ-9 score of 10 or greater were classified as having depression.

### 2.3. Data collection

The PHQ-9 depressive symptom assessment was conducted by the interviewer in a private face-to-face interview at the Mobile Examination Center (MEC). Questions about genital herpes and genital warts infection were answered in a private room of the MEC using an audio computer-assisted self-interview system.

### 2.4. Covariates

In this study, we selected demographic, social, and personal health factors associated with depression and STIs from the NHANES database to control for potential confounders.^[21,25–27]^ Demographic factors encompassed age, sex, and race. Social factors comprised educational attainment^[2,25,28,29]^ and poverty-to-income ratio (PIR).^[2,4,25,30]^ Personal health factors included alcohol use,^[4,25,31–33]^ waist circumference,^[34,35]^ body mass index (BMI),^[21,32,36]^ diabetes,^[21,36]^ stroke,^[37,38]^ asthma,^[37,39]^ hypertension,^[21,36,37]^ cancer,^[37,40]^ and coronary heart disease.^[21,35,36]^ The NHANES dataset also included additional racial/ethnic categories: non-Hispanic Whites, non-Hispanic Blacks, Mexican Americans, and others. Educational attainment was categorized as less than high school, high school or equivalent General Education Development, and above high school.

### 2.5. Statistical analysis

All analyses incorporated the NHANES complex survey design. We constructed 12-year MEC weights by dividing the 2-year examination weights (WTMEC2YR) by the number of combined cycles (n = 6) and specified these weights together with strata (SDMVSTRA) and primary sampling units (SDMVPSU); standard errors were obtained via Taylor series linearization. To address missing covariate data, we performed multiple imputation in IBM SPSS Statistics 27 using fully conditional specification, generated *m* = 20 imputed datasets, and pooled estimates via Rubin rules; each imputed dataset was analyzed using the complex samples procedures. Descriptive statistics and group comparisons used complex survey procedures, survey-weighted linear regression for continuous variables and the Rao–Scott χ² test for categorical variables, with 2-sided α = 0.05. Multivariable associations between depression status and STIs (genital herpes; genital warts) were estimated with survey-weighted logistic regression implemented in IBM SPSS Statistics 27 (Complex Samples module; CSDESCRIPTIVES/CSTABULATE for descriptives and CSLOGISTIC for models); figures were created in EmpowerStats 6.0. Three hierarchical models were fitted: Model 1 unadjusted; Model 2 adjusted for sociodemographic covariates (gender, age, race/ethnicity, educational attainment, and family income-to-poverty ratio); and Model 3 fully adjusted for demographic, socioeconomic, and personal health confounders. Stratified analyses were conducted across prespecified subgroups. As a sensitivity analysis, propensity score matching was performed via subclassification (seed = 40; caliper = 0.2 SD); the propensity model included all variables in Model 3, balance was assessed using standardized mean differences (|SMD|<0.10), and pre-/post-matching diagnostics are provided in Table S1–S6 and Figures S1 and S2, Supplemental Digital Content, https://links.lww.com/MD/R24. Post-match outcome models retained the NHANES design (12-year MEC weights, SDMVSTRA, SDMVPSU) and were estimated with complex samples procedures.

## 3. Results

### 3.1. Baseline characteristics of the participants

Table 1 summarizes the baseline characteristics of the 18,862 participants aged ≥20 years in the 2005 to 2016 NHANES. The weighted mean age was 39.2 years (95% CI: 38.8–39.5), with a balanced gender distribution (50.6% female, 95% CI: 49.8–51.3; 49.4% male, 95% CI: 48.7–50.2). Racial composition analysis revealed non-Hispanic whites as the predominant group (66.1%, 95% CI: 63.1%–69.0%), followed by non-Hispanic Blacks (11.8%, 95% CI: 10.3%–13.4%), Mexican Americans (9.4%, 95% CI: 8.0–11.1), other Hispanics (5.8%, 95% CI: 4.8%–6.9%), and other ethnic groups (6.9%, 95% CI: 6.2%–7.6%).

**Table 1:** Characteristics of participants aged ≥20 years according to whether suffering from genital herpes or genital warts.

Characteristic	Mean (95% CI) (N = 18862)	Whether suffering from genital herpes			Whether suffering from genital warts		
		Nongenital herpes (N = 18,141)	Genital herpes (N = 721)	*P*	Nongenital warts (N = 18,111)	Genital warts (N = 751)	*P*
Age (yr)	39.2 (38.8–39.5)	39.0 (38.7–39.4)	42.0 (40.7–43.3)	<.001	39.0 (38.7–39.4)	41.8 (40.9–42.7)	<.001
Ratio of family income to poverty	3.0 (2.9–3.1)	3.0 (2.9–3.1)	3.3 (3.1–3.4)	.002	3.0 (2.9–3.1)	3.3 (3.1–3.4)	<.001
Waist circumference (cm)	100.6 (100.0–101.1)	100.7 (100.1–101.2)	98.5 (96.5–100.5)	.026	100.6 (100.1–101.2)	99.3 (97.6–101.0)	.105
Gender (%)				<.001			<.001
Female	50.6 (49.8–51.3)	49.5 (48.7–50.2)	74.5 (70.4–78.2)		49.8 (49.0–50.6)	65.7 (61.1–70.1)	
Male	49.4 (48.7–50.2)	50.5 (49.8–51.3)	25.5 (21.8–29.6)		50.2 (49.4–51.0)	34.3 (29.9–38.9)	
Race/Ethnicity (%)				<.001			<.001
Mexican American	9.4 (8.0–11.1)	9.7 (8.2–11.4)	4.1 (2.6–6.3)		9.7 (8.2–11.4)	5.2 (4.1–6.6)	
Other Hispanic	5.8 (4.8–6.9)	5.8 (4.9–7.0)	4.6 (3.4–6.2)		5.9 (5.0–7.1)	3.1 (2.1–4.6)	
Non-Hispanic White	66.1 (63.1–69.0)	65.9 (62.8–68.8)	71.5 (66.9–75.6)		65.5 (62.4–68.4)	79.2 (75.7–82.3)	
Non-Hispanic Black	11.8 (10.3–13.4)	11.6 (10.1–13.3)	15.6 (12.7–19.0)		11.9 (10.4–13.6)	8.6 (6.7–11.0)	
Other race-including multi-racial	6.9 (6.2–7.6)	7.0 (6.3–7.8)	4.3 (3.0–6.1)		7.0 (6.3–7.8)	3.9 (2.5–6.1)	
Education level (%)				<.001			<.001
Less than high school	13.9 (12.7–15.2)	14.1 (12.9–15.4)	10.2 (7.7–13.4)		14.2 (13.0–15.5)	9.1 (6.8–12.0)	
High school or GED	21.1 (19.9–22.3)	21.3 (20.1–22.5)	16.6 (13.3–20.5)		21.3 (20.1–22.5)	17.6 (14.7–20.8)	
College or above	62.6 (60.6–64.6)	62.1 (60.1–64.1)	72.6 (67.8–77.0)		62.1 (60.0–64.0)	72.9 (68.8–76.7)	
NA	2.4 (2.2–2.7)	2.5 (2.2–2.8)	0.6 (0.2–1.6)		2.5 (2.2–2.8)	0.4 (0.1–1.3)	
Diabetes (%)				.049			.336
No	92.9 (92.4–93.4)	92.8 (92.3–93.3)	94.0 (91.9–95.6)		92.9 (92.4–93.4)	93.2 (90.1–95.3)	
Yes	5.5 (5.1–6.0)	5.6 (5.2–6.1)	3.6 (2.5–5.0)		5.6 (5.1–6.0)	4.5 (2.7–7.3)	
Borderline	1.5 (1.3–1.8)	1.5 (1.3–1.8)	2.4 (1.4–4.0)		1.5 (1.3–1.8)	2.3 (1.3–4.1)	
NA	0.0 (0.0–0.1)	0.0 (0.0–0.1)	0.0 (0.0–0.0)		0.0 (0.0–0.1)	0.1 (0.0–0.4)	
Alcohol use (%)				<.001			<.001
No	8.9 (8.0–9.9)	9.1 (8.1–10.1)	4.7 (3.3–6.5)		9.2 (8.2–10.2)	2.8 (1.8–4.3)	
Yes	10.4 (9.7–11.2)	10.5 (9.8–11.3)	7.6 (5.8–10.0)		10.6 (9.9–11.4)	6.1 (4.0–9.1)	
NA	80.7 (79.4–82.0)	80.4 (79.1–81.7)	87.7 (84.9–90.1)		80.2 (78.8–81.5)	91.1 (88.0–93.5)	
Body mass index (kg/m^2^) (%)				.171			.207
<25	26.6 (25.6–27.7)	26.6 (25.5–27.6)	27.9 (24.2–32.0)		26.5 (25.4–27.5)	29.4 (25.1–34.0)	
25–<30	27.1 (25.8–28.4)	26.9 (25.6–28.2)	30.9 (26.6–35.5)		27.0 (25.7–28.3)	29.2 (25.3–33.5)	
≥30	28.6 (27.4–29.9)	28.7 (27.5–30.0)	25.6 (21.5–30.2)		28.8 (27.5–30.0)	25.2 (21.5–29.2)	
NA	17.7 (16.0–19.6)	17.8 (16.1–19.6)	15.6 (10.9–21.7)		17.8 (16.1–19.6)	16.3 (11.9–21.8)	
Asthma (%)				.231			.857
No	84.3 (83.6–85.0)	84.4 (83.7–85.1)	81.8 (77.6–85.3)		84.3 (83.6–85.0)	84.0 (80.5–86.9)	
Yes	15.6 (14.9–16.4)	15.5 (14.8–16.2)	18.2 (14.7–22.4)		15.6 (14.9–16.3)	16.0 (13.1–19.4)	
NA	0.1 (0.0–0.2)	0.1 (0.0–0.2)	0.0 (0.0–0.0)		0.1 (0.0–0.2)	0.0 (0.0–0.3)	
Hypertension (%)				.776			.725
No	77.4 (76.4–78.5)	77.4 (76.3–78.4)	78.1 (74.1–81.7)		77.4 (76.4–78.4)	78.3 (74.3–81.8)	
Yes	22.5 (21.5–23.5)	22.5 (21.5–23.6)	21.9 (18.3–25.9)		22.5 (21.5–23.6)	21.7 (18.2–25.7)	
NA	0.1 (0.0–0.1)	0.1 (0.0–0.1)	0.0 (0.0–0.0)		0.1 (0.0–0.1)	0.0 (0.0–0.0)	
Cancer (%)				<.001			<.001
No	92.3 (91.8–92.9)	92.4 (91.8–92.9)	90.8 (87.3–93.4)		92.4 (91.8–92.9)	91.3 (88.1–93.7)	
Yes	5.2 (4.8–5.7)	5.0 (4.6–5.5)	8.6 (6.0–12.1)		5.0 (4.6–5.5)	8.3 (6.0–11.3)	
NA	2.5 (2.2–2.8)	2.6 (2.3–2.9)	0.6 (0.2–1.6)		2.6 (2.3–2.9)	0.4 (0.1–1.3)	
Stroke (%)				.073			.004
No	96.4 (96.0–96.7)	96.3 (96.0–96.6)	98.2 (96.5–99.0)		96.3 (95.9–96.6)	98.3 (96.9–99.0)	
Yes	1.2 (1.0–1.4)	1.2 (1.0–1.4)	0.8 (0.3–2.2)		1.2 (1.0–1.4)	1.3 (0.6–2.6)	
NA	2.4 (2.2–2.7)	2.5 (2.2–2.8)	1.1 (0.5–2.4)		2.6 (2.3–2.9)	0.4 (0.1–1.3)	
Coronary heart disease (%)				.006			.005
No	96.5 (96.1–96.8)	96.4 (96.1–96.7)	98.3 (96.9–99.1)		96.4 (96.1–96.7)	97.9 (95.9–99.0)	
Yes	1.0 (0.8–1.2)	1.0 (0.8–1.2)	1.1 (0.5–2.4)		1.0 (0.8–1.2)	1.6 (0.7–3.7)	
NA	2.5 (2.3–2.8)	2.6 (2.4–2.9)	0.6 (0.2–1.6)		2.6 (2.4–2.9)	0.5 (0.2–1.3)	

CI = confidence interval, GED = General Educational Development, NA = not applicable.

Participants with genital herpes were significantly older (42.0 years, 95% CI: 40.7–43.3) compared with the noninfected group (39.0 years, 95% CI: 38.7–39.4; *P* < .0001) and had a higher proportion of females (74.5% vs 49.5% in noninfected females; *P* < .0001). Males constituted 25.5% of the herpes group versus 50.5% in the noninfected group. Socioeconomically, the herpes group showed a higher PIR (PIR: 3.3 vs 3.0; *P* = .0019) and greater educational attainment (72.6% college-educated vs 62.1%; *P* < .0001). Health indicators revealed lower rates of diabetes (3.6% vs 5.6%; *P* = .0485) and alcohol use (7.6% vs 10.5%; *P* < .001) but higher cancer prevalence (8.6% vs 5.0%; *P* = .0003). Waist circumference was smaller in the herpes group (98.5 vs 100.7 cm; *P* = .0255), and fewer participants had coronary heart disease (1.1% vs 1.0%; *P* = .0060). No significant BMI differences were observed between groups (*P* = .1713).

Similarly, the genital warts group exhibited an older age profile (41.8 vs 39.0 years; *P* < .001) and a higher proportion of females (65.7% vs 49.8%; *P* < .0001). Socioeconomic patterns mirrored the herpes group, with elevated PIR (3.3 vs 3.0; *P* = .0009). Health outcomes included lower alcohol use (6.1% vs 10.6%; *P* < .001) and a significantly higher proportion of stroke-free individuals (98.3% vs 96.3%; *P* = .0036). Coronary heart disease-free participants were also more common in the warts group (97.9% vs 96.4%; *P* = .0045). No significant BMI differences were observed between groups (*P* = .2072).

### 3.2. Association between depression and the risk of genital herpes or genital warts

**Figure 2.**
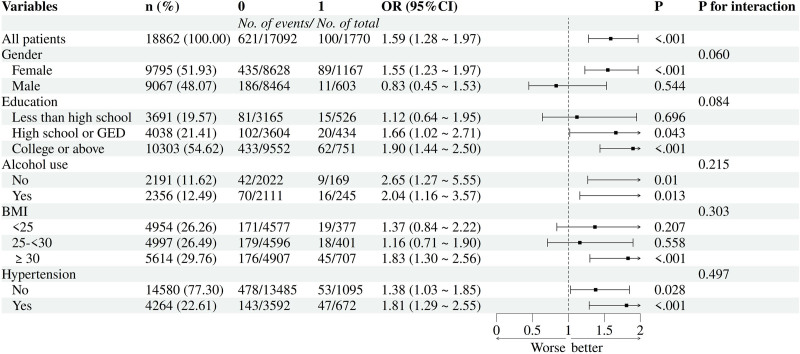
Forest plot of depression and risk for genital herpes.

**Figure 3.**
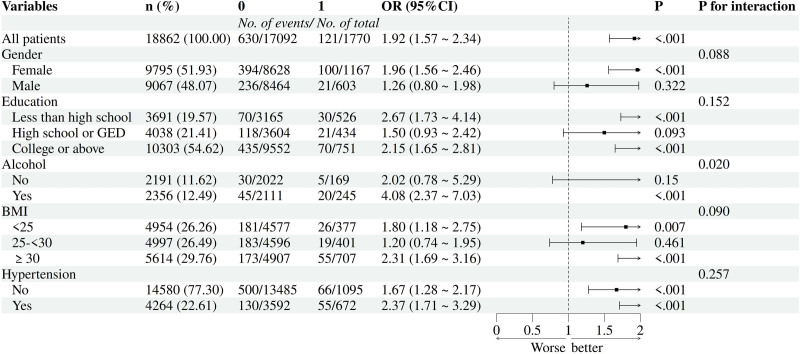
Forest plot of depression and risk for genital warts.

Multivariate logistic regression analyses were performed to assess associations between depression and 2 STIs: genital herpes and genital warts. As summarized in Table 2, depression consistently showed significant positive correlations with both outcomes across 3 analytical models. In the unadjusted baseline model (Model 1), odds ratios (ORs) were 1.80 (95% CI: 1.30–2.48; *P* < .001) for herpes and 1.78 (1.38–2.29; *P* < .001) for warts. Adjusting for sociodemographic factors (Model 2) slightly reduced effect sizes but retained significance (herpes: OR = 1.71, 1.22–2.41; warts: OR = 1.80, 1.36–2.37; *P* ≤ .002). Full adjustment for biomedical covariates in Model 3 yielded ORs of 1.73 (1.22–2.45; *P* = .003) and 1.79 (1.36–2.35; *P* < .001) for herpes and warts, respectively. Figures 2 and 3 present our bar graphs, which visually delineate the relationship between depression and the risk of these 2 STIs.

**Table 2:** Association between depression and genital herps/genital warts.

Characteristic	Genital herpes									Genital warts								
	Model 1			Model 2			Model 3			Model 1			Model 2			Model 3		
	OR	95% CI	*P*	OR	95% CI	*P*	OR	95% CI	*P*	OR	95% CI	*P*	OR	95% CI	*P*	OR	95% CI	*P*
Depression																		
No	—	—		—	—		—	—		—	—		—	—		—	—	
Yes	1.80	1.30–2.48	<.001	1.71	1.22–2.41	.002	1.73	1.22–2.45	.003	1.78	1.38–2.29	<.001	1.80	1.36–2.37	<.001	1.79	1.36–2.35	<.001
Gender																		
Female	1.66	1.19–2.34	.004	1.85	1.29–2.64	<.001	1.90	1.31–2.74	<.001	1.77	1.32–2.38	<.001	1.97	1.44–2.71	<.001	1.99	1.45–2.73	<.001
Male	1.07	0.51–2.25	.900	1.15	0.52–2.57	.700	1.04	0.45–2.41	.917	1.18	0.65–2.15	.600	1.28	0.70–2.34	.400	1.31	0.71–2.41	.400
Body mass index (kg/m²) (%)																		
<25	2.14	1.10–4.17	.026	2.10	1.05–4.19	.037	2.00	0.99–4.04	.053	1.80	1.08–3.01	.024	1.98	1.19–3.30	.010	1.96	1.17–3.29	.012
25–<30	0.81	0.43–1.50	.500	0.84	0.43–1.61	.600	0.85	0.43–1.67	.600	0.90	0.51–1.58	.700	0.98	0.53–1.81	.971	0.96	0.52–1.77	.900
≥30	1.96	1.29–3.00	.002	1.72	1.09–2.71	.021	1.72	1.08–2.74	.023	1.93	1.24–3.02	.004	1.65	1.03–2.65	.037	1.60	0.99–2.59	.057
Education level																		
Less than high school	0.99	0.46–2.15	.90	0.81	0.34–1.94	.600	0.73	0.33–1.61	.400	3.29	1.81–5.98	<.001	2.58	1.39–4.81	.003	2.34	1.37–3.99	.002
High school or GED	1.71	0.92–3.17	.087	1.45	0.78–2.67	.200	1.34	0.72–2.46	.300	1.33	0.73–2.44	.300	1.25	0.69–2.28	.500	1.14	0.63–2.09	.700
College or above	2.24	1.47–3.41	<.001	2.04	1.31–3.19	.002	2.21	1.39–3.50	.001	1.90	1.34–2.69	<.001	1.80	1.25–2.60	.002	1.86	1.29–2.68	.001
Hypertension																		
No	1.59	1.07–2.36	.023	1.56	1.04–2.34	.033	1.51	0.99–2.30	.055	1.61	1.20–2.16	.002	1.64	1.20–2.23	.002	1.53	1.10–2.12	.011
Yes	2.36	1.51–3.68	<.001	2.09	1.30–3.37	.003	2.02	1.23–3.31	.006	2.25	1.44–3.52	<.001	2.26	1.44–3.54	<.001	2.28	1.43–3.62	<.001
Alcohol use																		
No	2.37	1.05–5.38	.039	2.37	1.00–5.61	.049	2.28	0.89–5.84	.084	2.15	0.76–6.09	.150	2.00	0.65–6.10	.200	2.02	0.56–7.23	.300
Yes	1.97	1.04–3.73	.037	1.64	0.81–3.30	.200	1.69	0.83–3.45	.150	2.87	1.61–5.14	<.001	2.61	1.44–4.70	.002	2.41	1.34–4.34	.004

Model 1: The crude model.

Model 2: Gender, age, race, education level, and ratio of family income to poverty were adjusted.

Model 3: In addition to the adjustments made in Model 2, alcoholic drinks, BMI, waist circumference, diabetes, stroke, cancer, asthma, hypertension, and coronary heart disease were also adjusted.

BMI = body mass index, CI = confidence interval, GED = General Educational Development, OR = odds ratio.

Stratified analyses (Table 2) revealed gender-specific disparities, with women exhibiting stronger associations (all *P* < .001). Higher BMI (≥30) correlated with elevated herpes risk (*P* < .05), while lower BMI (<25) linked to increased warts likelihood (*P* < .05). Education levels also influenced outcomes: college-educated individuals showed heightened risks for both infections (*P* < .01), whereas those with less than high school education had elevated warts risk (*P* < .01). Hypertension independently predicted herpes (*P* < .01) and warts (*P* < .01), and alcohol use was tied to higher warts incidence (*P* < .01).

## 4. Discussion

This cross-sectional study analyzed data from the NHANES 2005 to 2016 cohort (adults aged ≥ 20 years) to examine the direct association between depression and risks of genital herpes and warts. Results demonstrated that US adults with depression exhibited significantly elevated risks for both infections (*P* < .05), with associations remaining statistically significant after full adjustment for confounders. Stratified analyses revealed heterogeneity across gender, BMI categories, education levels, and health profiles. Notably, female sex, postsecondary education, and hypertension emerged as shared risk factors for both conditions among individuals with depression.

The elevated infection risk in depression may stem from impaired impulse control, maladaptive coping strategies, reduced self-regulation, and increased high-risk sexual behaviors. Most depression patients exhibit cognitive deficits in attention, executive function, visuospatial learning, and memory,^[41,42]^ which impair impulse regulation.^[43]^ Severe cases may progress to impulse control disorders, driving repetitive harmful behaviors.^[44]^ Khan et al suggested that diminished impulse control reduces STI risk recognition and avoidance capacity in depression,^[45]^ while Leppink et al found severe depression linked to compulsive behaviors in college students.^[46]^ If impaired impulse control represents a passive risk factor, cognitive avoidance and low self-efficacy reflect active behavioral choices. Cognitive avoidance involves redirecting attention to pleasurable stimuli to evade negative emotions while disregarding socially adaptive norms.^[47]^ Low self-efficacy, a diminished perception of control over life events, correlates with depressive states and maladaptive avoidance.^[48,49]^ Alvy et al identified cognitive avoidance and self-efficacy as mediators between depression and risky sexual behaviors,^[47]^ while Braje et al proposed that such behaviors enable emotional disengagement, developing a cognitive avoidance model to assess risk in depressed individuals.^[50]^

Biological pathways may further explain these associations. Chronic inflammation, a low-grade inflammation in depression,^[51]^ may impair antiviral defenses by suppressing Th1-type immune responses through pro-inflammatory cytokines such as interleukin (IL)-6 and tumor necrosis factor (TNF)-α.^[52]^ Th1 responses are critical for combating intracellular pathogens such as viruses and *Mycobacterium tuberculosis*.^[53–55]^ Depressed individuals show elevated circulating IL-1, IL-6, and TNF-α levels,^[56,57]^ potentially weakening HSV and HPV clearance. Furthermore, hypothalamic-pituitary-adrenal (HPA) axis dysregulation contributes to this mechanism: cortisol abnormalities from HPA axis hyperactivity suppress CD8⁺ T-cell function, essential for controlling latent viral reactivation.^[58]^ Cortisol levels correlate positively with stress intensity and depression severity,^[59]^ while chronic stress-induced HPA hyperactivity has been shown to increase latent HSV-1 reactivation in animal models.^[60]^

Shared risk factors include sex, education, and hypertension. Regarding sex differences, females show heightened susceptibility, which may arise from 3 interrelated mechanisms: greater disease burden, evidenced by higher incidence rates compared with males^[27,61]^; behavioral risks associated with increased exposure through multiple male partners^[2,62,63]^; and diagnostic bias, as routine gynecological screenings enhance asymptomatic infection detection in women, whereas males lack standardized diagnostic protocols.^[11]^ Education level exhibits paradoxical associations with infection risk. Depression patients without high school education demonstrate elevated wart risk, likely due to limited health literacy and poor awareness of STI consequences.^[29,64]^ Conversely, individuals with higher education exhibit increased herpes/wart risk, potentially linked to riskier sexual behaviors within this demographic.^[62]^ Hypertension, which co-occurs with depression^[65]^ in 26.8% of cases,^[66]^ may indirectly elevate infection risk through shared pathophysiological pathways.

Previous epidemiological investigations have predominantly treated STIs as aggregated diagnostic entities, typically combining genital herpes (HSV) and HPV-associated warts with other STIs under composite clinical endpoints. In contrast, our study systematically disentangles these 2 conditions as distinct analytical variables, thereby uncovering both replicated and previously unrecognized associations with depression. This approach resolves a critical limitation in prior literature: although Huang et al’s seminal cohort work^[21]^ demonstrated elevated risks for multiple STIs (including HIV, syphilis, and chlamydia) among individuals with depression, particularly women, and subsequent studies by Jenkins et al^[67]^ and Shrier et al^[20]^ corroborated this broad association, none specifically addressed herpesvirus or papillomavirus infections through dedicated analyses. By incorporating population diversity enhancements, optimized statistical power, disease-specific methodological frameworks, and comprehensive multivariable adjustments, our findings provide robust confirmation of depression’s unique relationships with both conditions. Observed discrepancies across studies likely reflect methodological heterogeneity, including the restriction of STI-depression associations to specific demographic subgroups (e.g., Black males in earlier reports^[45]^) versus our population-wide gender-based categorization, as well as fundamental differences in STI classification protocols. Because the prevalence of genital herpes/warts in our data is low (≈3.4%), the odds ratios from logistic regression are close to risk ratios. Accordingly, the observed relative elevations correspond to small absolute risk differences at the population level, even though they remain clinically meaningful for targeted prevention.

Beyond behavioral and biological mechanisms, health-system and social determinants of diagnosis may also shape the observed patterns.^[68]^ Differential access to screening and frequency of gynecological visits can increase opportunities to detect asymptomatic or subclinical HSV/HPV infections, potentially contributing to the higher observed rates among women and better-educated individuals.^[69]^ Stigma and care-seeking behavior may further influence who is tested and diagnosed.^[70]^ Because our pooled dataset does not include harmonized measures of healthcare utilization or screening intensity, we cannot directly adjust for these factors; thus, diagnostic-opportunity bias remains a plausible alternative explanation alongside biological susceptibility.^[71]^

Some limitations warrant consideration. First, the cross-sectional design precludes the establishment of causal inferences between depression and genital herpes/warts, necessitating future validation through prospective cohort studies. Second, findings derived from a nationally representative US sample may lack generalizability to non-US populations with distinct sociocultural contexts. Third, genital herpes and warts were ascertained by self-report. Because both conditions are often asymptomatic and stigmatized, underreporting is likely and would tend to bias associations toward the null if nondifferential.^[2,21,67,72]^ However, differential misclassification by depression status is also plausible: individuals with depression may overreport due to increased healthcare contact and diagnosis, or underreport because of shame or social withdrawal, which could respectively inflate or attenuate the observed associations. Given the absence of universal laboratory confirmation in NHANES for these outcomes, we cannot precisely quantify the magnitude of misclassification. The use of audio computer-assisted self-interview likely reduces, but does not eliminate, reporting bias. We therefore interpret effect sizes cautiously and note that this limitation is inherent to NHANES behavioral self-report data.

## 5. Conclusion

In conclusion, this study demonstrates increased genital herpes and wart risks among US adults with depression, identifying female sex, postsecondary education, and hypertension as significant risk modifiers. Practical recommendations include implementing rapid HSV-2/HPV testing in psychiatric clinics, adopting standardized PHQ-9 screenings in STI care settings, and establishing cross-departmental EHR alert systems to improve care coordination. Prospective studies remain crucial to establish causality and inform targeted prevention strategies for high-risk subgroups.

## Acknowledgments

The authors thank the staff and the participants of the NHANES study for their valuable contributions. The authors also acknowledge the utilization of IBM SPSS Statistics 27 (IBM Corp.) for data organization, EmpowerStats 6.0 (X&Y Solutions) for conducting model regression analyses and image processing, and Deepseek-R1 (DeepSeek Inc.) for grammatical refinement and fluency enhancement of the final English manuscript. The authors would like to clarify that DeepSeek-R1 was exclusively used for language refinement and grammar correction in the preparation of this manuscript. It was not involved in any data analysis, interpretation, or formulation of conclusions. This usage is fully disclosed and complies with the journal’s policy on the involvement of AI tools in manuscript preparation.

## Author contributions

**Conceptualization:** Hongren Wu, Qiuhong Ye, Pengcheng Sun, Chuanhui Yan, Jianjun Zhang.

**Data curation:** Hongren Wu.

**Formal analysis:** Hongren Wu, Pengcheng Sun.

**Funding acquisition:** Hongren Wu, Chuanhui Yan, Jianjun Zhang.

**Investigation:** Hongren Wu.

**Methodology:** Hongren Wu, Qiuhong Ye, Pengcheng Sun.

**Project administration:** Jianjun Zhang.

**Software:** Hongren Wu.

**Supervision:** Chuanhui Yan, Jianjun Zhang.

**Writing – original draft:** Hongren Wu, Qiuhong Ye.

**Writing – review & editing:** Qiuhong Ye, Pengcheng Sun, Chuanhui Yan, Jianjun Zhang.

## Supplementary Material


