# Supplementary material for: Depression increases the risk of contracting genital herpes and genital wart in U.S. adults: A cross-sectional analysis
Source: Medicine (Baltimore). 2025 Dec 26;104(52):e46800. doi: 10.1097/MD.0000000000046800 (PMC12746943; doi:10.1097/MD.0000000000046800)
Supplement: Supplementary file 1 [file medi-104-e46800-s001.pdf]

Supplementary figure 1 Standardized mean differences before and after propensity score matching—genital herpes analysis.

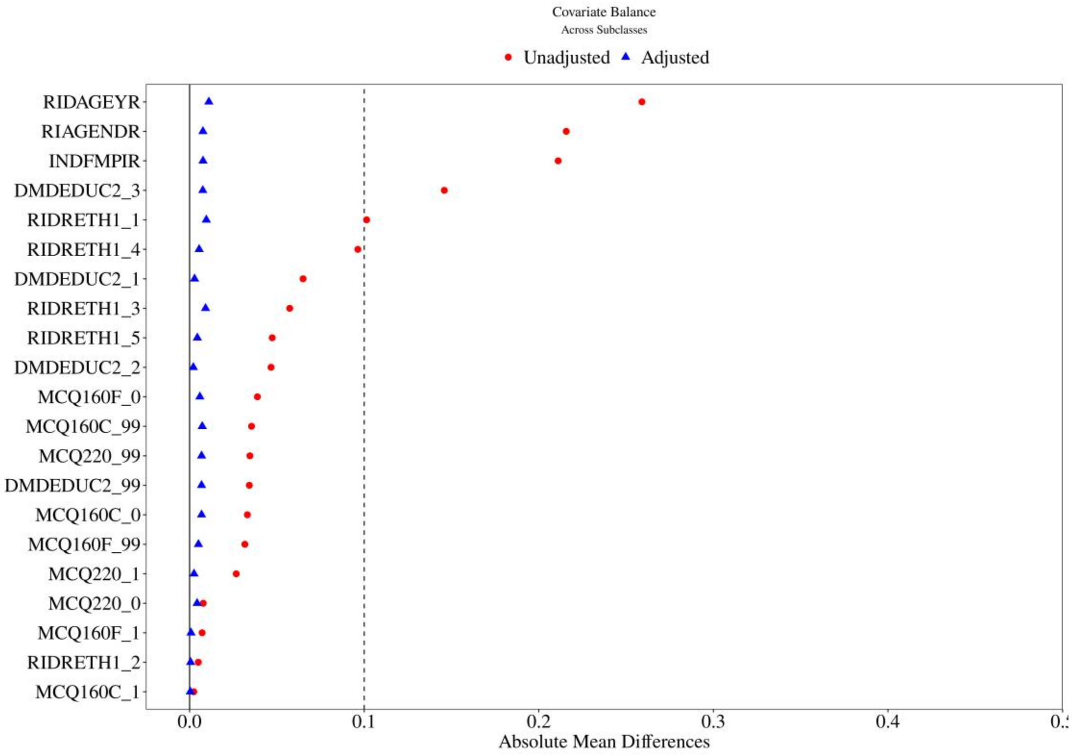

Supplementary figure 2 Standardized mean differences before and after propensity score matching—genital warts analysis.

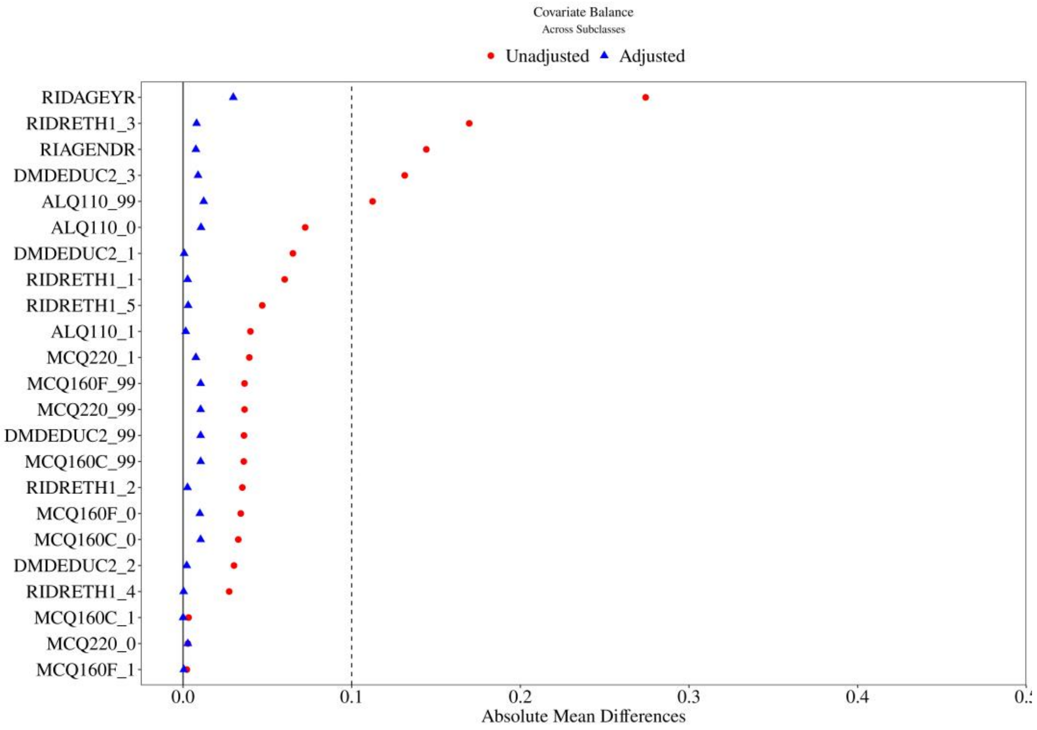

Supplementary table1:Before vs after propensity score matching—genital herpes analysis

Variable	Before PSM					After PSM				
	Total (n = 18862)	No (n = 18141)	Yes (n = 721)	SMD	*P*	Total (n = 1438)	No (n = 719)	Yes (n = 719)	SMD	*P*
Age, Mean ± SD	38.15 ± 11.84	38.05 ± 11.87	40.80 ± 10.65	0.259	<.001	40.65 ± 11.15	40.52 ± 11.64	40.77 ± 10.64	0.023	0.047
Waist Circumference Comment,Mean ± SD	100.45 ± 17.77	100.48 ± 17.79	99.83 ± 17.29	-0.037	0.339	99.99 ± 17.91	100.16 ± 18.53	99.82 ± 17.29	-0.020	0.167
Family PIR, M (Q□, Q□)	2.15 (1.06, 4.18)	2.14 (1.05, 4.13)	2.71 (1.26, 5.00)	0.211	<.001	2.31 (1.18, 4.22)	2.33 (1.19, 4.24)	2.88 (1.32, 5.11)	0.215	<.001
Gender, n (%)					<.001					0.033
Female	9795 (51.93)	9271 (51.11)	524 (72.68)	0.484		1046 (72.74)	524 (72.88)	522 (72.60)	0.006	
Male	9067 (48.07)	8870 (48.89)	197 (27.32)	-0.484		392 (27.26)	195 (27.12)	197 (27.40)	-0.006	
Race/Ethnicity, n (%)					<.001					0.046
Mexican American	3226 (17.1)	3173 (17.49)	53 (7.35)	-0.389		104 (7.23)	51 (7.09)	53 (7.37)	0.011	
Other Hispanic	1817 (9.63)	1751 (9.65)	66 (9.15)	-0.017		139 (9.67)	73 (10.15)	66 (9.18)	-0.034	
Non-Hispanic White	7802 (41.36)	7464 (41.14)	338 (46.88)	0.115		650 (45.2)	313 (43.53)	337 (46.87)	0.067	
Non-Hispanic Black	4112 (21.8)	3888 (21.43)	224 (31.07)	0.208		466 (32.41)	243 (33.80)	223 (31.02)	-0.060	
Other Race	1905 (10.1)	1865 (10.28)	40 (5.55)	-0.207		79 (5.49)	39 (5.42)	40 (5.56)	0.006	
Education Level, n (%)					<.001					<.001
Less than high school	3691 (19.57)	3595 (19.82)	96 (13.31)	-0.191		200 (13.91)	104 (14.46)	96 (13.35)	-0.033	
High school or GED	4038 (21.41)	3916 (21.59)	122 (16.92)	-0.124		239 (16.62)	117 (16.27)	122 (16.97)	0.019	
College or above	10303 (54.62)	9808 (54.07)	495 (68.65)	0.314		981 (68.22)	488 (67.87)	493 (68.57)	0.015	
NA	830 (4.4)	822 (4.53)	8 (1.11)	-0.327		18 (1.25)	10 (1.39)	8 (1.11)	-0.027	
Diabetes, n (%)					0.018					0.044
No	17317 (91.81)	16655 (91.81)	662 (91.82)	0.000		1314 (91.38)	652 (90.68)	662 (92.07)	0.051	
Yes	1250 (6.63)	1211 (6.68)	39 (5.41)	-0.056		83 (5.77)	45 (6.26)	38 (5.29)	-0.044	
Borderline	284 (1.51)	264 (1.46)	20 (2.77)	0.080		41 (2.85)	22 (3.06)	19 (2.64)	-0.026	
NA	11 (0.06)	11 (0.06)	0 (0.00)	-0.025		-	-	-	-	
Alcohol Drinks, n (%)					<.001					0.077
No	2191 (11.62)	2140 (11.80)	51 (7.07)	-0.184		100 (6.95)	49 (6.82)	51 (7.09)	0.011	
Yes	2356 (12.49)	2270 (12.51)	86 (11.93)	-0.018		182 (12.66)	96 (13.35)	86 (11.96)	-0.043	
NA	14315 (75.89)	13731 (75.69)	584 (81.00)	0.135		1156 (80.39)	574 (79.83)	582 (80.95)	0.028	
Body mass index(kg/m^2),n (%)					0.616					0.604

Variable	Before PSM					After PSM				
	Total (n = 18862)	No (n = 18141)	Yes (n = 721)	SMD	*P*	Total (n = 1438)	No (n = 719)	Yes (n = 719)	SMD	*P*
<25	4954 (26.26)	4764 (26.26)	190 (26.35)	0.002		368 (25.59)	178 (24.76)	190 (26.43)	0.038	
25-<30	4997 (26.49)	4800 (26.46)	197 (27.32)	0.019		396 (27.54)	200 (27.82)	196 (27.26)	-0.012	
≥30	5614 (29.76)	5393 (29.73)	221 (30.65)	0.020		435 (30.25)	215 (29.90)	220 (30.60)	0.015	
NA	3297 (17.48)	3184 (17.55)	113 (15.67)	-0.052		239 (16.62)	126 (17.52)	113 (15.72)	-0.050	
Asthma, n (%)					0.079					0.589
No	15899 (84.29)	15311 (84.40)	588 (81.55)	-0.073		1168 (81.22)	580 (80.67)	588 (81.78)	0.029	
Yes	2948 (15.63)	2815 (15.52)	133 (18.45)	0.076		270 (18.78)	139 (19.33)	131 (18.22)	-0.029	
NA	15 (0.08)	15 (0.08)	0 (0.00)	-0.029		-	-	-	-	
Hypertension, n (%)					0.036					0.673
No	14580 (77.3)	14049 (77.44)	531 (73.65)	-0.086		1069 (74.34)	538 (74.83)	531 (73.85)	-0.022	
Yes	4264 (22.61)	4074 (22.46)	190 (26.35)	0.088		369 (25.66)	181 (25.17)	188 (26.15)	0.022	
NA	18 (0.1)	18 (0.10)	0 (0.00)	-0.032		-	-	-	-	
Cancer, n (%)					<.001					0.531
No	17281 (91.62)	16615 (91.59)	666 (92.37)	0.030		1334 (92.77)	669 (93.05)	665 (92.49)	-0.021	
Yes	745 (3.95)	698 (3.85)	47 (6.52)	0.108		86 (5.98)	40 (5.56)	46 (6.40)	0.034	
NA	836 (4.43)	828 (4.56)	8 (1.11)	-0.330		18 (1.25)	10 (1.39)	8 (1.11)	-0.027	
Stroke, n (%)					<.001					0.775
No	17765 (94.18)	17059 (94.04)	706 (97.92)	0.272		1408 (97.91)	703 (97.77)	705 (98.05)	0.020	
Yes	261 (1.38)	256 (1.41)	5 (0.69)	-0.086		10 (0.7)	5 (0.70)	5 (0.70)	0.000	
NA	836 (4.43)	826 (4.55)	10 (1.39)	-0.271		20 (1.39)	11 (1.53)	9 (1.25)	-0.025	
Coronary Heart Disease, n (%)					<.001					0.047
No	17817 (94.46)	17113 (94.33)	704 (97.64)	0.218		1397 (97.15)	695 (96.66)	702 (97.64)	0.064	
Yes	192 (1.02)	183 (1.01)	9 (1.25)	0.022		23 (1.6)	14 (1.95)	9 (1.25)	-0.063	
NA	853 (4.52)	845 (4.66)	8 (1.11)	-0.339		18 (1.25)	10 (1.39)	8 (1.11)	-0.027	

Supplementary table 2:Before vs after propensity score matching—genital warts analysis

Variable	Before PSM					After PSM				
	Total (n = 18862)	No (n = 18141)	Yes (n = 721)	SMD	*P*	Total (n = 1502)	No (n = 751)	Yes (n = 751)	SMD	*P*
Age, Mean ± SD	38.15 ± 11.84	38.04 ± 11.89	40.87 ± 10.33	0.274	<.001	40.85 ± 11.20	40.83 ± 12.02	40.87 ± 10.33	0.004	0.042
Waist Circumference Comment,Mean ± SD	100.45 ± 17.77	100.46 ± 17.79	100.25 ± 17.28	-0.012	0.749	100.37 ± 17.65	100.49 ± 18.02	100.25 ± 17.28	-0.014	0.790
Family PIR, M (Q□, Q□)	2.15 (1.06, 4.18)	2.14 (1.06, 4.14)	2.54 (1.12, 4.86)	0.134	<.001	2.23 (1.14, 4.22)	2.23 (1.14, 4.22)	2.77 (1.24, 5.01)	0.144	<.001
Gender, n (%)					<.001					0.039
Female	9795 (51.93)	9301 (51.36)	494 (65.78)	0.304		983 (65.45)	489 (65.11)	494 (65.78)	0.014	
Male	9067 (48.07)	8810 (48.64)	257 (34.22)	-0.304		519 (34.55)	262 (34.89)	257 (34.22)	-0.014	
Race/Ethnicity, n (%)					<.001					0.141
Mexican American	3226 (17.1)	3141 (17.34)	85 (11.32)	-0.190		163 (10.85)	78 (10.39)	85 (11.32)	0.029	
Other Hispanic	1817 (9.63)	1770 (9.77)	47 (6.26)	-0.145		95 (6.32)	48 (6.39)	47 (6.26)	-0.005	
Non-Hispanic White	7802 (41.36)	7369 (40.69)	433 (57.66)	0.343		855 (56.92)	422 (56.19)	433 (57.66)	0.030	
Non-Hispanic Black	4112 (21.8)	3968 (21.91)	144 (19.17)	-0.069		320 (21.3)	176 (23.44)	144 (19.17)	-0.108	
Other Race	1905 (10.1)	1863 (10.29)	42 (5.59)	-0.204		69 (4.59)	27 (3.60)	42 (5.59)	0.087	
Education Level, n (%)					<.001					0.046
Less than high school	3691 (19.57)	3591 (19.83)	100 (13.32)	-0.192		194 (12.92)	94 (12.52)	100 (13.32)	0.024	
High school or GED	4038 (21.41)	3899 (21.53)	139 (18.51)	-0.078		280 (18.64)	141 (18.77)	139 (18.51)	-0.007	
College or above	10303 (54.62)	9798 (54.10)	505 (67.24)	0.280		1013 (67.44)	508 (67.64)	505 (67.24)	-0.009	
NA	830 (4.4)	823 (4.54)	7 (0.93)	-0.376		15 (1)	8 (1.07)	7 (0.93)	-0.014	
Diabetes, n (%)					0.014					0.434
No	17317 (91.81)	16630 (91.82)	687 (91.48)	-0.012		1368 (91.08)	681 (90.68)	687 (91.48)	0.029	
Yes	1250 (6.63)	1208 (6.67)	42 (5.59)	-0.047		96 (6.39)	54 (7.19)	42 (5.59)	-0.070	
Borderline	284 (1.51)	263 (1.45)	21 (2.80)	0.082		36 (2.4)	15 (2.00)	21 (2.80)	0.048	
NA	11 (0.06)	10 (0.06)	1 (0.13)	0.021		2 (0.13)	1 (0.13)	1 (0.13)	0.000	
Alcohol Drinks, n (%)					<.001					0.049
No	2191 (11.62)	2156 (11.90)	35 (4.66)	-0.344		61 (4.06)	26 (3.46)	35 (4.66)	0.057	
Yes	2356 (12.49)	2291 (12.65)	65 (8.66)	-0.142		136 (9.05)	71 (9.45)	65 (8.66)	-0.028	
NA	14315 (75.89)	13664 (75.45)	651 (86.68)	0.331		1305 (86.88)	654 (87.08)	651 (86.68)	-0.012	
Body mass index(kg/m^2),n (%)					0.390					0.959

Variable	Before PSM					After PSM				
	Total (n = 18862)	No (n = 18141)	Yes (n = 721)	SMD	*P*	Total (n = 1502)	No (n = 751)	Yes (n = 751)	SMD	*P*
<25	4954 (26.26)	4747 (26.21)	207 (27.56)	0.030		420 (27.96)	213 (28.36)	207 (27.56)	-0.018	
25-<30	4997 (26.49)	4795 (26.48)	202 (26.90)	0.010		395 (26.3)	193 (25.70)	202 (26.90)	0.027	
≥30	5614 (29.76)	5386 (29.74)	228 (30.36)	0.013		458 (30.49)	230 (30.63)	228 (30.36)	-0.006	
NA	3297 (17.48)	3183 (17.57)	114 (15.18)	-0.067		229 (15.25)	115 (15.31)	114 (15.18)	-0.004	
Asthma, n (%)					0.201					0.201
No	15899 (84.29)	15283 (84.39)	616 (82.02)	-0.061		1221 (81.29)	605 (80.56)	616 (82.02)	0.033	
Yes	2948 (15.63)	2814 (15.54)	134 (17.84)	0.060		279 (18.58)	145 (19.31)	134 (17.84)	-0.020	
NA	15 (0.08)	14 (0.08)	1 (0.13)	0.015		2 (0.13)	1 (0.13)	1 (0.13)	-0.042	
Hypertension, n (%)					0.280					0.593
No	14580 (77.3)	14014 (77.38)	566 (75.37)	-0.047		1123 (74.77)	557 (74.17)	566 (75.37)	0.028	
Yes	4264 (22.61)	4079 (22.52)	185 (24.63)	0.049		379 (25.23)	194 (25.83)	185 (24.63)	-0.028	
NA	18 (0.1)	18 (0.10)	0 (0.00)	-0.032		-	-	-	-	
Cancer, n (%)					<.001					0.044
No	17281 (91.62)	16595 (91.63)	686 (91.34)	-0.010		1365 (90.88)	679 (90.41)	686 (91.34)	0.033	
Yes	745 (3.95)	687 (3.79)	58 (7.72)	0.147		120 (7.99)	62 (8.26)	58 (7.72)	-0.020	
NA	836 (4.43)	829 (4.58)	7 (0.93)	-0.379		17 (1.13)	10 (1.33)	7 (0.93)	-0.042	
Stroke, n (%)					<.001					0.893
No	17765 (94.18)	17033 (94.05)	732 (97.47)	0.218		1461 (97.27)	729 (97.07)	732 (97.47)	0.025	
Yes	261 (1.38)	249 (1.37)	12 (1.60)	0.018		26 (1.73)	14 (1.86)	12 (1.60)	-0.021	
NA	836 (4.43)	829 (4.58)	7 (0.93)	-0.379		15 (1)	8 (1.07)	7 (0.93)	-0.014	
Coronary Heart Disease, n (%)					<.001					0.542
No	17817 (94.46)	17084 (94.33)	733 (97.60)	0.214		1468 (97.74)	735 (97.87)	733 (97.60)	-0.017	
Yes	192 (1.02)	182 (1.00)	10 (1.33)	0.028		16 (1.07)	6 (0.80)	10 (1.33)	0.046	
NA	853 (4.52)	845 (4.67)	8 (1.07)	-0.351		18 (1.2)	10 (1.33)	8 (1.07)	-0.026	

Supplementary table 3: Univariable logistic regression results—genital herpes analysis

Variables	β	S.E	Z	*P*	OR (95%CI)
Depression					
No					1.00 (Reference)
Yes	0.46	0.11	4.18	<.001	1.59 (1.28 ~ 1.97)

OR: Odds Ratio, CI: Confidence Interval

Supplementary table 4: Multivariable logistic regression results—genital herpes analysis

Variables	β	S.E	Z	*P*	OR (95%CI)
Intercept	-4.57	0.21	-21.67	<.001	0.01 (0.01 ~ 0.02)
Depression					
No					1.00 (Reference)
Yes	0.42	0.12	3.62	<.001	1.52 (1.21 ~ 1.91)

OR: Odds Ratio, CI: Confidence Interval

Supplementary table 5: Univariable logistic regression results—genital warts analysis

Variables	β	S.E	Z	*P*	OR (95%CI)
Depression					
No					1.00 (Reference)
Yes	0.65	0.10	6.35	<.001	1.92 (1.57 ~ 2.34)

OR: Odds Ratio, CI: Confidence Interval

Supplementary table 6: Multivariable logistic regression results—genital warts analysis

Variables	β	S.E	Z	*P*	OR (95%CI)
Intercept	-4.24	0.19	-22.08	<.001	0.01 (0.01 ~ 0.02)
Depression					
No					1.00 (Reference)
Yes	0.61	0.11	5.69	<.001	1.85 (1.50 ~ 2.28)

OR: Odds Ratio, CI: Confidence Interval
